# Study on extraction and characterization of anchote (*Coccinia abyssinica*) starch and reinforced enset (*Ensete ventricosum*) fiber for the production of reinforced bioplastic film

**DOI:** 10.1016/j.heliyon.2023.e23098

**Published:** 2023-12-01

**Authors:** Samuel Latebo Majamo, Temesgen Abeto Amibo

**Affiliations:** a Department of Chemical Engineering, College of Engineering and Technology, Wachemo University, Hossana, Ethiopia; b Department of Process Engineering and Chemical Technology, Faculty of Chemistry, Gdansk University of Technology, Narutowicza 11/12, 80-233, Gdansk, Poland; c School of Chemical Engineering, Jimma Institute of Technology, Jimma University, Jimma, P.O. Box-378, Ethiopia

**Keywords:** Anchote starch, Enset fiber, Reinforced plastic film, Tensile strength, Elongation

## Abstract

Population expansion is causing an increase in dependence on plastic materials. The worst aspects of conventional plastics were their inability to biodegrade, their poor capacity to transmit water vapor, and their production of greenhouse gases. Usages of bioplastics are necessary for the advancement of a green economy and environment in order to eradicate these drawbacks of traditional plastics. In this study, reinforced bioplastic film was produced from anchote *(Coccinia Abyssinica)* starch and enset (*Ensete Ventricosum*) fiber. Starch from anchote was extracted and its properties were characterized via adequate techniques. The maximum carbohydrate content (86.26 ± 0.25%w/w) of anchote starch indicates that it is suitable feedstock for plastic film production. In addition, extracted starch was characterized by SEM, FTIR, TGA and XRD. The reinforcing material enset fiber was extracted and characterized by FTIR and XRD. The results of both feedstock materials exhibited the good characteristics and viability for bioplastic film production. Enset fiber loadings used were 0 %, 4 %, 8 %, 12 % and 16 % w/w in starch basis. Tensile strength, elongation, thickness, moisture content, transparency, solubility and density of produced bioplastic were determined. Tensile force grew and elongation reduced as fiber loading rose up to 8 %. The tensile strength gradually declined with increasing fiber loading. Additionally, the created bioplastic film's groups of functions and chemical bonds were examined. In comparison to unreinforced plastic film, the results showed that the reinforced bioplastic film used in this study was an excellent and effective product.

## 1 Background of the study

The biggest challenge confronting humanity now is achieving sustainable growth without damaging the environment. Therefore, the environment and equitable development are now important topics [1,2]. Synthetic plastics are being replaced with biodegradable polymers, specifically those made from renewable resources [[3], [4], [5]].

When opposed compared to traditional plastics, using bioplastics have many benefits. Over their lifetime, bioplastics emit fewer greenhouse gases than conventional plastics. Consequently, bioplastics help to create a society that is more sustainable. It is eco-friendly and biodegradable. Bioplastics are essential in a variety of application domains. They are more valuable since they have a smaller environmental impact when compared to conventional polymers. However, their lack of mechanical strength restricts their use. Although they are not biodegradable, carbon and glass fibers are artificial fibers that are frequently utilized to strengthen bioplastics. Therefore they can be replaced with readily available, affordable, and sustainable materials, called lignocellulosic fibers [6,7].

Biomass material is essential to maintaining a sustainable environment and necessary to produce useful material for a variety of applications as renewable and eco-friendly materials gain popularity. Plants materials create a range of renewable polymers, including cellulose, starch, and protein. Renewable biopolymers may be produced with resources that are continuously supplied, which makes them more sustainable than conventional plastics. Flexible coatings and stiff packaging are just two applications for bioplastics [5].

The primary sources of starch in the globe include corn, potatoes, wheat, cassava, and sweet potatoes. Due to the overuse of the traditional sources of starch, new botanical sources of starch must be researched [3]. Due to its wide range of applications, suitable alternative should be employed for innovative crops and plant components with maximum starch content. Ethiopia is regarded to be the origin and diversity of many crop species in Africa. *Coccinia abyssinica*, *Ensete veaniricosum*, *Plectranthus edulis* (Ethiopian potato), and other notable indigenous tuber crops are cultivated in diverse parts of Ethiopia. The cultivation of native Ethiopian tuber crops is now generally recognized due to their economic and nutritional advantages. Using accessible resources to produce starch [8,9]. It is beneficial to produce starch from inexpensive local feedstocks [10,11].

The macromolecules can be altered by a variety of processes and are created by nature or through chemical reactions derived from sources that are naturally occurring [12,13]. However, when these natural polymers were processed separately, the majority of them displayed poor mechanical qualities and dimensional consistency, which led to highly fragile products [10]. One solution to this problem is to incorporate reinforcement substances, such as natural fibers, into the matrix of polymers [14]. As compared to fiber derived from various fibrous agricultural waste products, such as pseudostem, fruit stalk, and leaf, enset fiber has the highest cellulose percentage as well as important physical properties [[15], [16], [17]] [[15], [16], [17]] [[15], [16], [17]]. Fiber derived from natural plants is essential for making rainforced biomaterial composites and food packaging [8]. According to recent research, enset fiber has significant chemical and physical qualities that make it a valuable raw material for packaging industries [18].

The intention behind this study is to synthesis natural resource-based biodegradable plastic to replace traditional chemical polymer in food wrapping. In this study, anchote starch and enset fiber were employed. In this study, glycerol was utilized as a plasticizer and cellulosic fiber was added as reinforcement to an anchote starch matrix to create an environmentally acceptable bioplastic. Evaluation of the chemical, mechanical, and physical qualities was done with care. The results of the experiment showed that developed biosynthesized plastic sheet exhibited desirable characteristics.

## 2 Materials and methods

### 2.1 Materials

#### 2.1.1 Raw materials

The primary raw components used to produce bioplastic film were starch and fiber. The anchote plant's starch was extracted. Enset cellulosic fiber was employed as reinforcement for this product. Anchote and enset fiber were collected from locally accessible locations in Hossana city's countryside, Ethiopia.

#### 2.1.2 Chemicals

There were various reagents and chemicals employed when conducting present experiments. All of them were graded. All necessary chemicals’ obtained company was Kirkos Shopping Center C-322. The principal chemicals employed in this investigation were; sodium bisulfate is used as a preserving agent, and enset fiber is treated with 0.3 M NaOH (99.9 % purity) to treat and remove it of undesirable components. Glycerol (99.8 % purity) used as a plasticizer, hydrogen peroxide for bleaching, HCl (99.9 %), H_2_SO_4_ (98 %), and catalyst tablet VST (cod. A00000277) were used to determine protein.

#### 2.1.3 Equipments and instruments

Equipments used in this experiments include a weighing scale (OHAUS CORP-ARA520), a temperature-adjustable hot plate (STUART UC125), oven (DHG-9055A), a hot air oven (OV/125/SS/F/DIG/A), a muffle furnace (S30 2RR, England), spectrophotometer (UV 7804C), scanning electron microscopy (FEI INSPECT F50 model), powder X-ray diffractometer (XRD-7000), Thermogravimetric Analysis (TA instrument, model: SDT Q600), Fourier transform infrared spectroscopy (Spectrum 65 FT-IR, PerkinElmer), and others numerous equipments were used during conducting the experiments. Mechanical tests (tensile strength and elongation) were performed by applying ASTM D3039 procedure using universal testing machine.

### 2.2 Experimental framework

**Fig. 1:**
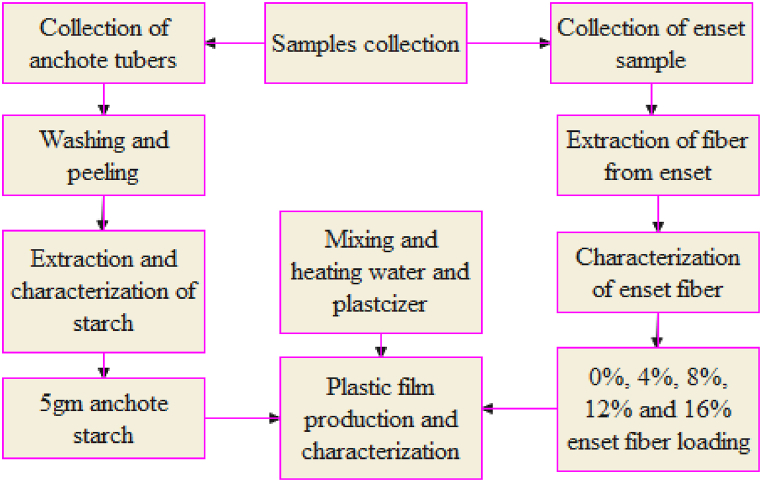
General experimental framework of the current study.

The experimental frame work of this study was generalized as shown in Fig. 1. The overall structure for this study covered everything from the gathering of samples to synthesize and determination of physical mechanical and chemical characteristics of bioplastic film.

### 2.3 Starch extraction from anchote tubers

Anchote starch was extracted by modifying the method described [9]. To get rid of the dirt on the surface, fresh anchote tubers were carefully rinsed in distilled water. After that, the fruit was peeled and cut into 1 cm cubes using a conical juice mixer. Starch was extracted using distilled water and anchote peel at a ratio of 1:2 (g/mL). For the purpose of removing any remaining proteins and phenolic chemicals, the pulped material was suspended in excess 0.02 % NaOH. The upper part was discarded and the starch was collected. The resultant starch collected was dried for 12 h at 40 °C in an oven drier. Finally, a laboratory grinding device was used to ground the dried starch into a fine powder. Finally, isolated starch was characterized and then used as main raw material blinding with enset fiber for bioplastic film production.

### 2.4 Extraction of enset fiber

The enset fiber was extracted and treated by modifying the method sugested by Ref. [19]. Using an iron ribbon, the fiber was removed by rubbing the enset tissue. Subsequently, the fiber went through a 4-h soak at 90 °C in a 2 wt percent (2 wt%) of sodium hydroxide (NaOH) solution. For the alkali treatment, the fiber to liquid mixture was 1:25 (g/mL). After that, it was cleaned with distilled water and proceeded to dry for 48 h at room conditions.

### 2.5 Characterization of starch

#### 2.5.1 Chemical composition

The official methods of AOAC (2005) were used to examine starch composition: moisture content (950.46), ash (942.05), total protein (984.13), crude ﬁber (962.09), and carbohydrates content, it was determined by subtracting the total percentage of other components from 100.

#### 2.5.2 Crystallinity of starch

The XRD instrument was employed to determine the starch crystallinity. The diffractometer was in 2θ mode of operation. The device ran at 30 Kv of voltage and 25 mA of current. An analysis of the XRD peaks and pattern were conducted at room conditions.

#### 2.5.3 FTIR characterization of anchote starch

The functional components that were accessible and the entire bonding in the material were discovered using FTIR spectroscopy. Samples were grinded into fine powder and mixed with potassium bromide that was used as reference material. A ray of light was focused on the sample at a spatial resolution of 4 cm^−1^, capturing the spectrum of the infrared bands within the 400–4000 cm^−1^ range. The available components were analyzed.

#### 2.5.4 Thermogravimetric Analysis (TGA)

TGA was applied to determine the weight degradation profile of fiber at degradation temperature. The heating rate was 20 °C/min. The weight losses of the fiber with corresponding degradation temperature were recorded and analyzed.

#### 2.5.5 Scanning electron microscopy (SEM) analysis

The picture of anchote starch was generated using a SEM (FEI INSPECT F50 model). Molecules and atoms in the samples interact to generate a multitude of signals, containing information about the exterior morphology of the sample. It was talked about the SEM image in result and discussion section.

### 2.6 Characterization of enset fiber

#### 2.6.1 FTIR analysis of enset fiber

FTIR spectroscopy of the same model used for starch characterization was used in fiber analysis. Extracted enset fibers’ functional groups and entire bonds determined.

#### 2.6.2 X-ray diffractometer pattern of enset fiber

Similar XRD instrument which was used in starch characterization also used to characterize the fiber.

### 2.7 Preparation of bioplastics film

The bioplastic film was prepared by the procedure adopted from the method described by Ref. [13]. 100 ml of distilled water and 5 g of extracted starch were combined to create a film-forming dispersion. The dispersion was heated to 75 °C in a water bath and agitated for 20 min to gelatinize it. Extracted enset fiber was mixed with samples using HCl (3.5 mL) and glycerol (3 mL) while utilizing the same amount of starch in varying ratios (0 %, 4 %, 8 %, 12 %, 16 %, and 20 % w/w of starch). Following the observation of homogeneity, each mixture was mixed and allowed to cool to 48 °C. A Petri dish was used to create bioplastic sheets. Samples were chilled at 35 °C. Analysis was done on the manufactured bioplastic films.

### 2.8 Characterization of bioplastics film

#### 2.8.1 Water solubility test

The procedure used to determine solubility of bioplastic was adopted from literature [19]. Two-centimeter-by-two-centimeter film disks were cut, weighed (W_1_), and placed in a beaker with 50 ml of distilled water. The sample pieces were removed after 24 h of gentle agitation at 25 °C and dried for 24 h in an air-circulating oven at 105 °C until constant weight (W_2_) was attained. Triplicate experiments were conducted. The average value of solubility of bioplastic film was determined using equation (1).

(1) $$\begin{array}{l} \text{Water}\;\text{solubilty}\;\left(\%\right)=\frac{W_{1}-W_{2}}{W_{1}}*100\;\\ \text{Where};\;W_{1}=\text{initial}\;\text{mass}\;\text{and}\;W_{2}=\text{final}\;\text{mass} \end{array}$$

#### 2.8.2 Water absorption test

Bioplastic film's water absorption was tested using the established procedure (ASTM D570-98 2000). Bioplastic sheets were shrunk to a size of around 2 × 2 cm^2^. For 24 h, the bioplastic sheets were placed in a Petri plate with distilled water. 24 h later. The produced bioplastic weight was measured and noted. Two more experiments were conducted. The average value of water absorption was calculated by using equation (2).

(2) $$\begin{array}{l} \text{Water}\;\text{absorption}\;\left(\%\right)=\frac{W_{w}-W_{d}}{W_{w}}*100\;\\ \text{Where};\;W_{w}=\text{wet}\;\text{weight}\;\text{and}\;W_{d}=\text{dry}\;\text{weight} \end{array}$$

#### 2.8.3 Transparency of bioplastic film

Using a spectrophotometer (UV 7804C), the transparency of the bioplastic sheet was evaluated. According to Z. Assefa, 2013 [20], the bioplastic film transmittance was measured at 600 nm. Two more experiments were conducted. Average transparency in percentage was determined using equation (3).

(3) $$\begin{array}{l} \text{Transparency}\left(\%T\right)=\frac{-\text{logT}600\text{nm}}{X}\;\\ \text{Where}\;T600=\text{is}\;\text{the}\;\text{transmittance}\;\text{at}\;600\text{nm}\;\text{and}\;X\;\text{is}\;\text{the}\;\text{film}\;\text{thickness}\;\left(\text{mm}\right)\text{.} \end{array}$$

#### 2.8.4 The thickness of films

The film thickness was measured using digital micrometers and measurements were recorded. Triplicate experiments were conducted. Average film thickness was determined.

#### 2.8.5 Tensile strength and elongation at break

The standard technique (ASTM D3039) used in literatures [13,21] was utilized to determine tensile strength and elongation testing. Texture analyzer was used to investigate these features. The test specimens were preconditioned for 40 h at 23 °C and 50 % relative humidity. Then, 100 mm by 10 mm-sized film specimens were created. The crosshead speed of the texture analyser was set at 50 mm/min. Tensile strength was calculated in Mega Pascal (MPa), and at break, elongation was calculated in percent using equation (4) and equation (5) respectively. Triplicate experiments for elongation and tensile strength for each test were conducted. The average value of each test was taken.

(4) $$\text{Tensile}\;\text{strength}\;\left(\text{MPa}\;\right)=\frac{\text{Force}\;\left(N\right)}{\left(\text{width}*\text{thickness}\right){\text{mm}}^{2}}$$

(5) $$\text{Elongation}\;\left(\%\right)=\frac{\text{Increase}\;\text{in}\;\text{length}\left(\text{mm}\right)}{\text{Original}\;\text{length}\left(\text{mm}\right)}*100$$

#### 2.8.6 FTIR analysis of the plastic film

The chemical bonds and functional groups of bioplastic were identified by FTIR. The FTIR instrument of the same model was used for starch and fiber characterization analysis.

## 3 Result and discussion

### 3.1 Characterizations result of anchote tubers starch

#### 3.1.1 Chemical composition of starch

The chemical composition of anchote starch and comparative literatures result were presented in Table 1. The moisture content of anchote starch was 6.5 ± 0.12 %, which is relatively lowest content compared to moisture content of babassu mesocarp starch [22] and corn starch [23] as shown in Table 1. This indicates the process handled in good manner. Starch having low moisture content cannot affect the plastic film production. The ash content of anchote starch in present study was 0.15 ± 0.02 %. which was the reasonable agreement with other plant starch ash content [9,24]. The lower ash content may suggest that the low mineral content is good for further processing. The value of crude fiber was 3.78 % reasonably higher than values obtained in literatures [22,23]. The high fiber content is important to increase the strength of bioplastic. The Carbohydrate content present study was 86.26 ± 0.25 %.Which is slightly lower than other carbohydrate content plants. This is due the composition of carbohydrate in anchote plant species. For the synthesizing of bioplastic film, it is advised to use plant starch with high carbohydrate content [23,24].

**Table 1:** Chemical composition of native anchote starch and other natural plants starch.

Natural plants	Parameters					References
	Ash (%)	Moisture (%)	Total protein (%)	Crude fiber (%)	Carbohydrate (%)	
Anchote starch	0.15 ± 0.02	6.5 ± 0.12	3.17 ± 0.11	3.87 ± 0.09	86.26 ± 0.25	(Present study)
Potato starch	0.33 ± 0.15	4.03 ± 0.21	0.20 ± 0.05	–	89.30 ± 0.43	[9]
White rice starch	0.21 ± 0.01	4.76 ± 0.59	3.89 ± 0.09	–	90.75 ± 0.58	[24]
Babassu mesocarp	1.13 ± 0.12	15.09 ± 1.65	1.38 ± 0.01	1.72 ± 0.1	94.02 ± 0.12	[22]
Corn starch	0.14 ± 0.02	12.30 ± 0.16	0.74 ± 0.09	0.85 ± 0.08	97.73 ± 0.53	[23]

#### 3.1.2 FTIR analysis of native anchote starch

**Fig. 2:**
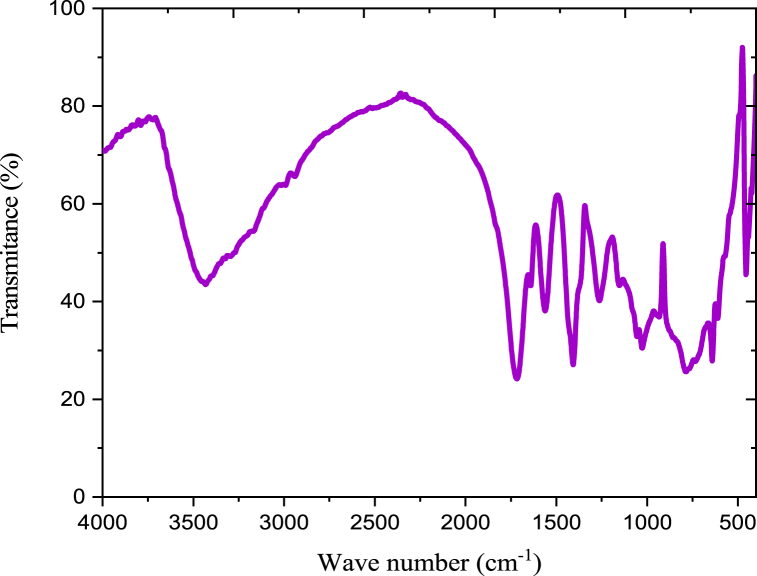
FT-IR spectra of anchote starch.

The FT-IR spectrum of anchote starch was presented in Fig. 2. The broad strong band at 3420 cm^−1^ represents the stretching vibrations of O–H group [22]. The peaks at 1647 cm-1 indicate that O–H is being bent by the adsorbed water molecules of H–*O*–H. At peak 1420 and 1383 cm^−1^, CH_2_ and C–H groups and bonds were assigned respectively. The C–H vibrations assigned to the O–H groups at peak 2921 cm-1. In anchote starch, the absorption band at 1153 cm^−1^ indicates to the coupling of C–O bonds. In starch molecule the absorption peaks at1080 cm^−1^ related to C–*O*–H bonds. Additional absorption peaks at 930, 856, 650, 583, and 530 cm^−1^ are related with C–*O*–C glycosidic linkage ring [22,23].

#### 3.1.3 XRD analysis of native anchote starch

**Fig. 3:**
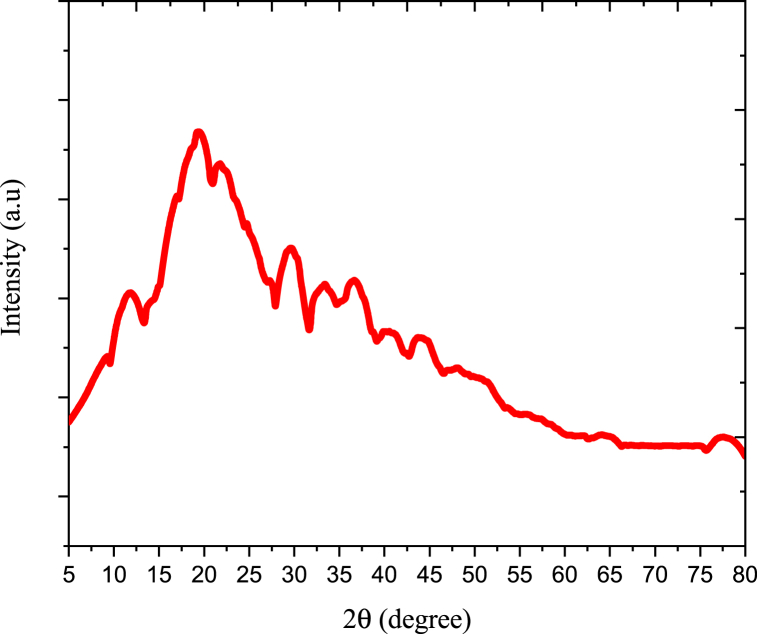
X-ray diffraction of native anchote starch.

Fig. 3 displayed the XRD pattern analysis of anchote starch. The organization of the double helical amylopectin chain is shown by the diffraction pattern. A, B, and C-type starches were used to depict the starch x-ray patterns [25]. Strong diffraction peaks for A-type cereal starch may be found at 15° and 23° of 2θ, as well as twice at 17° and 18° of 2θ. Strong peaks at 17° of 2θ, distinctive peaks at 5.6° of 2θ, and weak peaks at 5.81° of 2θ distinguish B-type plant starches. The B-type and A-type polysaccharides are combined to form C-type starch patterns. The strong diffraction peaks at 17° of 2θ with other characteristic peaks at 17.21°, 19.74°, 22.31°, 24.08° 38.7°, 45.3° and 78.7° indicate the starch type is B-type starch. Due to the genetic diversity of the anchote species, the diffraction pattern of the current trials showed a slight change from the prior ones [9,10].

#### 3.1.4 TGA analysis of anchote starch

**Fig. 4:**
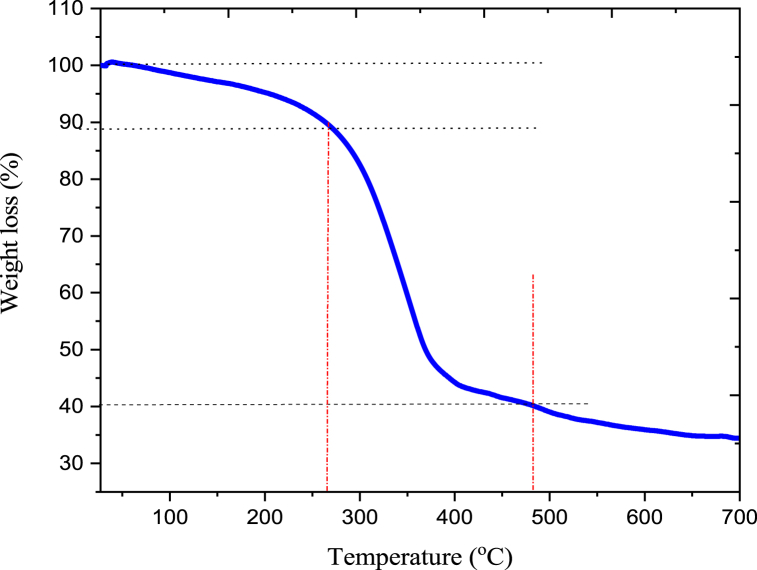
TGA curves of native anchote starch.

The thermogravimetric analysis profile is shown in Fig. 4. Three steps were shown in the weight reduction process. In the temperature range of 29–265 °C, physical adsorbed matter, volatile substances, and hydrogen-bonded water were seen in starch. The second weight loss happened between 265 and 483° Celsius. The TGA curve shows that the highest deterioration took place between 265 and 483 °C. Water is a byproduct of the inter- and intermolecular dehydration processes. The starch degrades as a result. The third weight loss was noticed in the 483–700 °C temperature range. This results in the decomposition of monomers and the generation of ash. Anchote starch's thermal behavior was demonstrated to be consistent with earlier research [9,10].

#### 3.1.5 Morphological analysis of native anchote starch

**Fig. 5:**
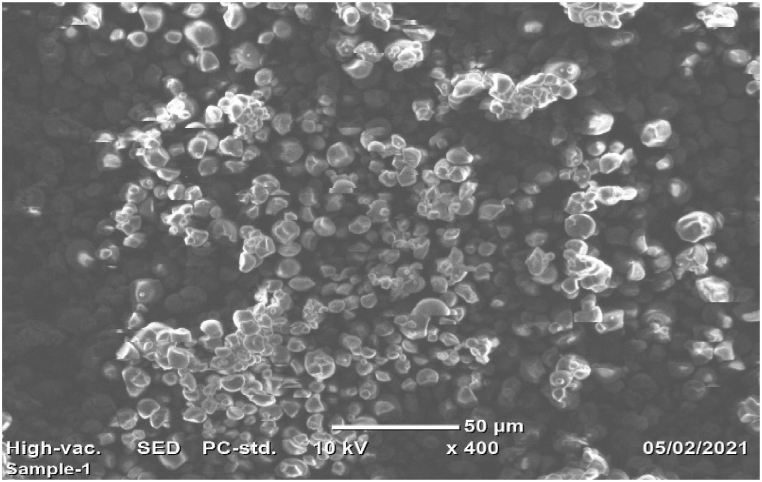
SEM image of anchote starch.

SEM was used to analyze the morphological features of the anchote starch granules. The morphological traits of the anchote starch granules are depicted in Fig. 5's SEM picture. The microscopic structure of the granules of anchote starch that have been discovered differs slightly from that described in earlier studies [11]. According to SEM findings, the dome-shaped, polygonal, and occasionally irregularly shaped granules of anchote starch were presented. This was so because the physiology and biological origin of the plant have an impact on the shape and structure of the starch granules. The mean width, length, and size of the anchote starch granules were 9.23, 11.57, and 3.15–19.99 μm, respectively.

### 3.2 Characterization of enset fiber

#### 3.2.1 FTIR spectroscopy analysis of enset fiber

**Fig. 6:**
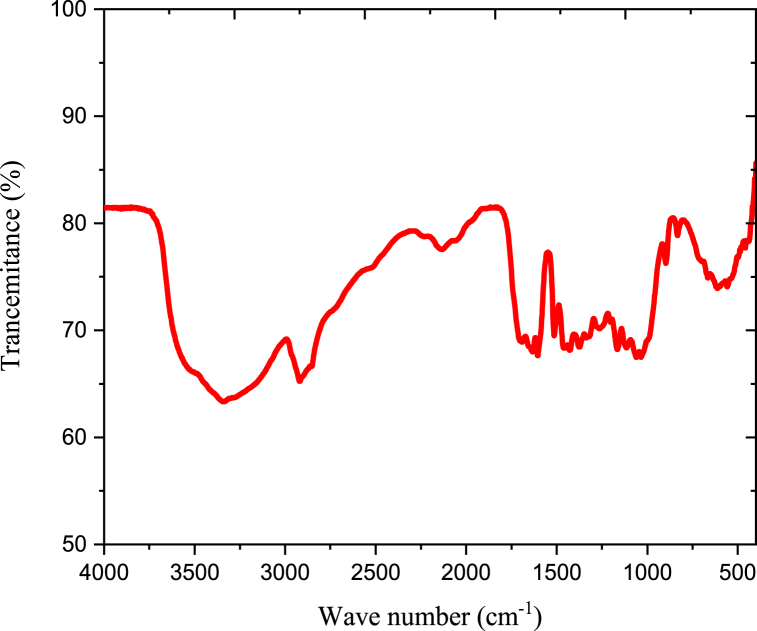
FTIR spectra of extracted cellulosic enset fiber.

Fig. 6 displayed the enset fiber's FTIR data. The absorption spectrums of enset fiber reveal information on the structure, chemical bonds, and functional groups of the carbon skeleton [26]. Hemicellulose, lignin, and cellulose are the three primary parts of the fiber shown in Fig. 6. Strong stretching of O–H bands at 3404 cm^−1^, aliphatic chain C–H symmetric stretching at 2910 cm^−1^, C–H absorption peaks at 1376 cm^−1^, and C–*O*–C glycosidic linkages at 1160 cm^−1^ were all associated with cellulose. the 1736 cm^−1^ absorption band connected to the 1250 cm^−1^ stretching vibration of the C=O group [27]. It was discovered that lignin has three absorption bands at 1603, 1514, and 1048 cm^−1^, which are related to secondary alcohols' C–O deformations [28].

Aromatic skeletal resonance breathing uses aliphatic ethers and C=O stretching, respectively. The secondary alcohols of lignin's C–O deformation are shown by the absorption band at 1603 cm^−1^. Further bands were found at 1513 and 1047 cm^−1^, which were connected to lignin's aliphatic ethers and aromatic structural oscillation with C=O stretching, respectively [29].

#### 3.2.2 XRD analysis of enset fiber

**Fig. 7:**
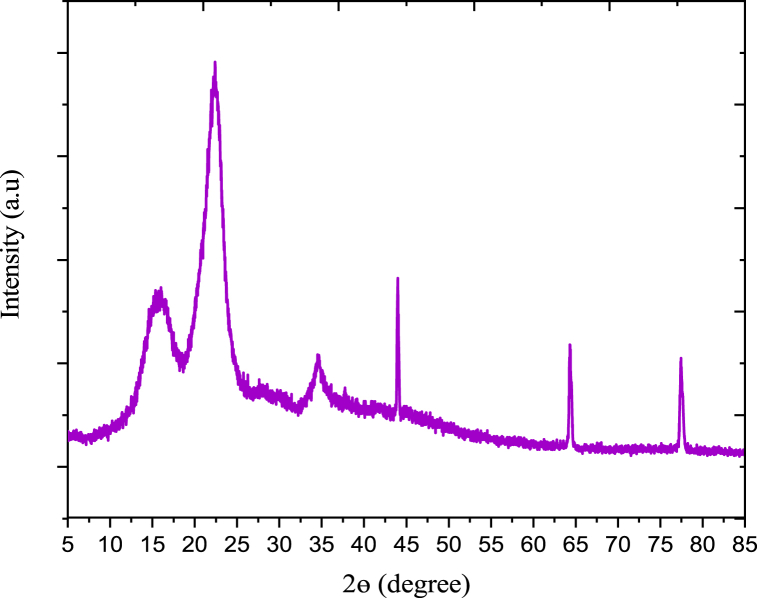
XRD pattern of enset fiber.

The XRD pattern of enset fiber was presented in Fig. 7. An enset cellulosic fiber exhibited reflection peaks at 2θ = 15.5°, 22.3°, 34.5°, 45.1°, 65.3° and 77.9°. These peaks are the characteristic of the crystal form of cellulose [30]. In the crystallographic planes of cellulosic fibers, the three distinctive peaks were visible. The enset fiber's XRD pattern reveals that it was mostly composed of cellulose with minor levels of hemicellulose and lignin. In fact, the XRD patterns of enset fiber demonstrate the applicability of reinforcing material in the manufacturing of plastic for enhancing stiffness and brightness [31].

### 3.3 Physical and mechanical properties of the bioplastics film

Table 2, involved that the physical and mechanical properties as tensile strength, moisture content, transparency, elongation at break, thickness, solubility and density of bioplastic film with different enset fiber loading were determined. Thickness and density of bioplastic film increased with increasing the fiber loading. The moisture content, transparency and solubility were decreasing with increasing fiber loading. This is due to the reinforcing material has the ability to setup the moisture content then after increases the thickness and density of the film. These results were good agreement with literature [32].

**Table 2:** Properties for both reinforced and control films of present study.

Fiber loading, % w/w of starch	Tensile strength (MPa)	Elongation (%)	Thickness (mm)	Moisture (%)	Transparency (%)	Solubility (%)	Density(g/ml)
0 %	6.54 ± 0.33	19.89 ± 0.32	0.410 ± 0.23	9.55 ± 0.21	115.22 ± 0.23	46.24 ± 0.35	1.64 ± 0.26
4 %	7.88 ± 0.18	18.46 ± 0.15	0.414 ± 0.17	9.01 ± 0.22	113.32 ± 0.21	43.95 ± 0.31	1.67 ± 0.19
8 %	8.34 ± 0.18	17.65 ± 0.21	0.416 ± 0.14	8.56 ± 0.28	110.43 ± 0.17	42.05 ± 0.25	1.69 ± 0.16
12 %	7.55 ± 0.17	16.91 ± 0.25	0.418 ± 0.13	8.34 ± 0.24	105.15 ± 0.19	41.15 ± 0.31	1.72 ± 0.22
16 %	6.88 ± 0.34	16.86 ± 0.29	0.421 ± 0.31	8.32 ± 0.32	104.95 ± 0.18	40.55 ± 0.24	1.73 ± 0.17

#### 3.3.1 Investigation of elongation at break and tensile strength

**Fig. 8:**
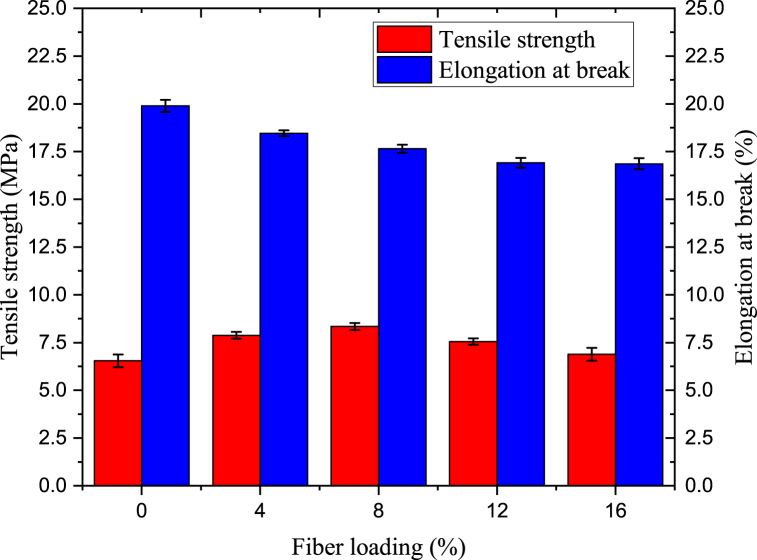
The effect of enset fiber loading on elongation at break and tensile strength of bioplastic film.

The *ensete ventricosum* fiber reinforcement of production bio plastic film has increases as shown in Fig. 8. The results showed as the growth of tensile strength with the fiber loading equal to 8 %. However, the tensile strength slowly decreased when fiber loading beyond 8 %. The decrease in tensile strength at greater fiber loading could be attributed to filler dispersion imperfections at higher loading. The bioplastic film (pure) with tensile strength of 6.54 MPa was increased to 7.88 by reinforcing 4 % fiber. The tensile strength of bioplastic film was 8.34 MPa when the fiber loading was 8 %. This fiber loading leads to maximum tensile strength. With the increase of fiber loading decreases the elongation at break. The percent elongation at break were19.89, 18.46, 17.65, 16.91 and 16.86 % when the fiber loading 0, 4, 8, 12, and 16 % w/w respectively. This decrement elongation at break was due to the fiber loading, which promote brittleness and increase rigidity of the reinforced bio plastic film [32].

#### 3.3.2 Fourier transform infrared spectrometry (FTIR) analysis of the film

**Fig. 9:**
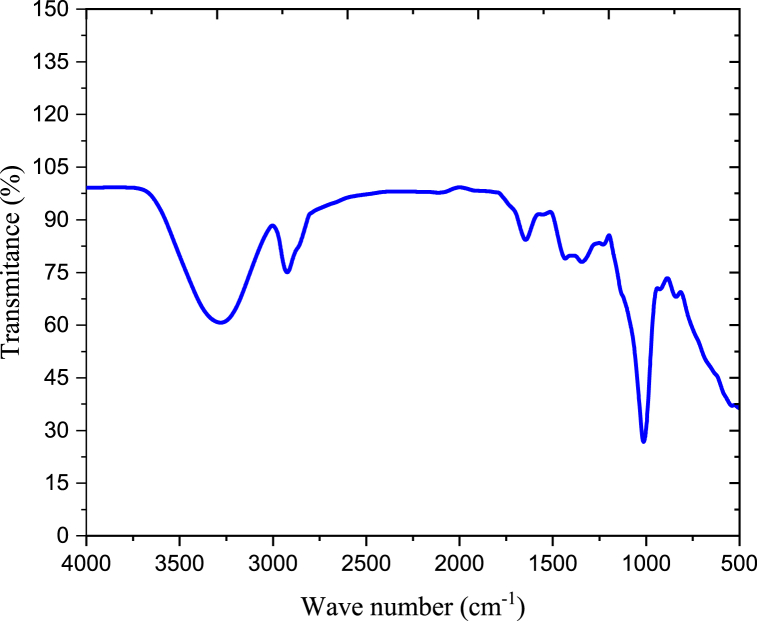
FT-IR spectra of bioplastic film.

The FT-IR spectra of cellulosic fiber-based films produced from anchote starch and enset cellulosic fiber are displayed in Fig. 9. The band generally vibrated in the manner described. 3010-2824.6 cm^−1^ assigned to C–H stretches. Absorption spectra at 1150 cm^−1^ linked to C–C stretching, 1235-1150 cm^−1^ related to C–*O*–C stretching of ester, 1734-1631 cm^−1^ related to bound water, and 3300-3400 cm^−1^ related to -*O*-H stretching because of polymeric involvement of hydroxyl groups are all connected to C–H stretches [33]. Hydrogen bonding between the CH_2_–OH groups of the starch and cellulose fiber are possible [4]. By generating or being reinforced by the starch matrix, the fiber appears to favor starch based on their chemical relationship [33].

### 3.4 Synthesized bioplastic film properties and standard food packaging materials’ properties

It is critical to compare and examine the properties of the bioplastic film synthesized in this study with the acceptable properties and standard values of food packaging materials. Food packaging materials must have recyclable properties, a writable surface, flexibility, and stability. These qualities were observed in the bioplastic film produced in this work. As a result, it is suitable for food packing. Furthermore, the translucency of traditional food packaging material is within the 85–90 % range [34]. Transparency can be greater than 90 % in modified packaging materials. This is an acceptable value for food packaging materials. Transparency of 8 % fiber loading produced bioplastic film is 110 %. This is an acceptable value. The tensile strength and density of the bioplastic synthesized in this investigation were approximated to those of ordinary plastic polymers' tensile strength and densities reported in literature [35]. Furthermore low moisture content of bioplastic film of this study suggests that it is suitable for food packaging.

## 4 Conclusion

In current study enset cellulosic fiber was used as reinforcing agent in bioplastics film production. In addition, anchote starch was used as starting material for synthesizing of bioplastics. The starch extracted from anchote was characterized by FTIR spectroscopy, SEM, XRD and TGA. The reinforcing material, enset fiber was characterized by FTIR and XRD. The characteristics of starch and fiber were more suitable for bioplastic film production. An enset fiber loading were used as 0 %, 4 %, 8 %, 12 % and 16 % w/w by starch basis. Physical and mechanical features of synthesized bioplastic were analyzed. The fiber loading increases to 8 %, by increasing tensile strength and decreasing elongation. The fiber loading increases by decreasing tensile strength. The attached components of the bioplastic film were identified and interpreted. There was efficient relation between anchote starch and enset fiber for the synthesis of biodegradable bioplastics. All in all cellulosic fiber from enset was good reinforcing agent for bioplastic production, because it exhibited good physiochemical and mechanical characteristics.

## 5 Recommendation

In this study some of basic properties were conducted and compared with standards of food packaging materials. However, it is better if future researchers who interested in this research area carryout fully thermal, mechanical and biological properties of bioplastic film and compare to standards of food packaging materials.

## Data availability

All data involved in this manuscript is available. So that me as first and corresponding author, at any necessary time, I can provide required data.

## CRediT authorship contribution statement

**Samuel Latebo Majamo:** Writing - review & editing, Writing - original draft, Validation, Methodology, Investigation, Formal analysis, Data curation, Conceptualization. **Temesgen Abeto Amibo:** Writing - review & editing, Validation.

## Declaration of competing interest

The authors declare that they have no known competing financial interests or personal relationships that could have appeared to influence the work reported in this paper.
